# Severe lumbar pain in an adolescent due to idiopathic vena cava thrombosis: a case report

**DOI:** 10.1186/s13256-023-03866-5

**Published:** 2023-05-12

**Authors:** Anthony Gevers, Patricia Dessart, Jean-Marie Vanmarsenille, Bruno Vande Berg, Halil Yildiz, Samar M. Hatem

**Affiliations:** 1 Department of Physical and Rehabilitation Medicine, Centre Hospitalier Valisana, Brussels, Belgium; 2 Faculty of Medicine, Université Catholique de Louvain, Brussels, Belgium; 3 Department of Physical and Rehabilitation Medicine, Cliniques Universitaires Saint-Luc, Brussels, Belgium; 4 Department of Radiology, Cliniques Universitaires Saint-Luc, Brussels, Belgium; 5 Department of Radiology, Centre Hospitalier Chrétien (CHC) Montlegia Clinic, Liege, Belgium; 6 Department of Internal Medicine and Infectious Diseases, Cliniques Universitaires Saint-Luc, Brussels, Belgium; 7 Institute of Neuroscience (IoNS), Université Catholique de Louvain (UCLouvain), Brussels, Belgium; 8 Department of Physical and Rehabilitation Medicine, Universitair Ziekenhuis Brussel (UZ Brussel), Brussels, Belgium; 9 STIMULUS Consortium (reSearch and TeachIng neuroModULation Uz bruSsel), Vrije Universiteit Brussels, Brussels, Belgium

**Keywords:** Case reports, Vena cava, Inferior, Thrombosis, Adolescent, Low back pain, Red flag

## Abstract

**Background:**

Lumbar back pain in an adolescent is generally musculoskeletal, that is, due to a muscle strain or scoliosis. This case describes a young individual without any previous health issues who rapidly developed a life-threatening condition, though initially presenting with “only” back pain.

**Case presentation:**

A 16-year-old Caucasian male patient was admitted twice to the emergency department with debilitating lumbar pain without neurological or vascular symptoms. Imagery showed an extensive thrombosis of the inferior vena cava. No external cause, structural abnormality, or any systemic disease were found that predisposed the patient to this highly unusual vaso-occlusive incident.

**Conclusion:**

Thrombosis of the inferior vena cava is a rare but life-threatening entity. It is underrecognized and associated with serious short- and long-term morbidity and mortality. Increased awareness is essential because deep vein thrombosis in children seems to cause atypical symptoms, such as spinal pain or the absence of edema of the lower limbs, as in the present case.

## Background

Acute severe lumbar pain in patients younger than 20 years is considered to be a red flag in the management of low back pain [1] and should therefore not be dismissed as self-limiting. A thorough history taking, clinical examination, and diagnostic workup is vital, especially in pediatric patients. The cause of lumbar back pain in adolescents is most of the time of musculoskeletal origin, such as muscle strain [2]. Nevertheless, spinal pain in children remains a red flag *per se*, which warrants complementary investigations, especially in case of deteriorated health status. Common causes to exclude are scoliosis, spondylolysis, spondylolisthesis, ankylosing spondylitis, degenerative disk disease and herniation, and, rarely, malignancy [2]. In the present case report, elaborate biological testing and diagnostic imagery for all of these etiologies were negative.

## Case presentation

Here, we report the case of a 16-year-old young Caucasian man suffering from lumbar pain that irradiated to the right thigh and was associated with painful urination and right testicle tenderness. There was no previous history of medical or surgical illness, or traveling outside of Belgium. The patient was admitted to the emergency room in January 2020, three weeks after the start of the symptoms. Physical examination, urinalysis, and abdominal echography were negative. Blood analysis showed slightly elevated inflammatory parameters [white blood cell count (WBC) 10.47 × 10^9^/L; C-reactive protein 44.9 mg/L]. Paracetamol, tramadol, and diclofenac were prescribed for pain management. Diazepam was prescribed as a muscle relaxant. The patient was discharged.

The patient’s clinical situation worsened at home, leading to severely impaired stance and gait. One week after the first admission, the patient was readmitted to the emergency room. Physical examination was unremarkable except for a positive straight leg rising test at 15° on the right side. No red flags were retained (despite the patient’s young age). No further technical investigations were performed. The patient was discharged on the same day and an appointment was scheduled 1 week later at the outpatient clinic of physical medicine and rehabilitation.

Upon examination by the specialist in physical medicine and rehabilitation (5 weeks after initial symptoms), the patient was markedly pale and described shooting pains in both legs in lumbar flexion. He presented with camptocormia and decreased walking perimeter. Urogenital issues had disappeared. Palpation and percussion of the lumbar spine were painless. Neurological examination showed sharp and symmetrical osteotendinous reflexes of the lower extremities and a normal plantar flexion reflex. Straight leg rising test was positive at 30° bilaterally. Sensory testing for touch was normal. Despite high levels of pain (especially in the calves when walking on tiptoe), muscle strength was considered as normal in the lower extremities (motor research council scale: 5/5).

**Fig. 1:**
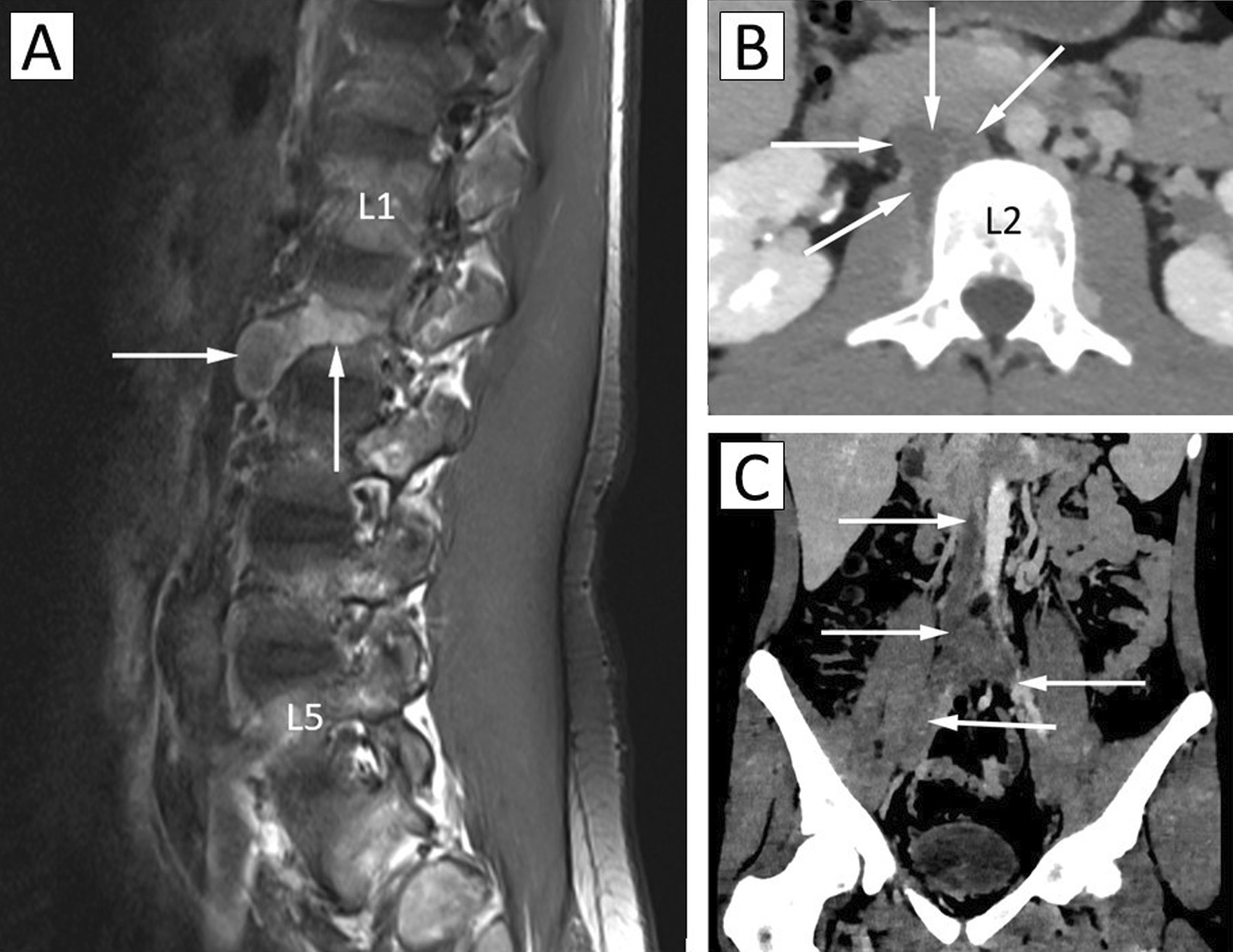
**A** Lumbar MRI (T1-weighted sagittal image). White arrows show the formation of a vascular venous abnormality adjacent to the L2 vertebral body in the laterovertebral right area, measuring 60 mm in its longest axis. **B** Abdominal CT (MonoE 50 keV—axial view). White arrows show a thrombus in the right L2 paravertebral vein. **C** Abdominal CT (with contrast—portal phase—MonoE 50 keV—coronal view). White arrows highlight the thrombosis of the inferior vena cava and two iliac veins

A septic discitis was suspected and a whole spine magnetic resonance imaging (MRI) was performed (Fig. 1A). No abnormalities of the lumbar spine were found. However, a formation of vascular venous aspect was observed in the laterovertebral right area adjacent to the L2 vertebral body, long axis measuring 60 mm, with flow anomaly and increased signal on T1- and T2-weighted images. At the level of L2, there appeared to be a narrowing of the lumen of the inferior vena cava with a thickening of the adjacent tissue-like wall (thickened by 6 mm on a 30 mm height). It was noted that this thickening encompassed the lower duodenum genu. The patient was hospitalized for further management.

Abdominal computed tomography angiography with contrast (Fig. 1B, C) highlighted a thrombosis of the infrarenal inferior vena cava extending to the iliofemoral veins with thrombosis of a right paralumbar vein in L2. The suprarenal vena cava was permeable but thin. No points of venous compression were seen. Multiple anterior retroperitoneal and parietal venous collaterals (epigastric) were present, suggesting a certain chronicity of the lesion.

Doppler echography of the lower extremities showed the presence of endovascular echogenic material in the entire deep venous network of both lower limbs (femoropopliteal and infrapopliteal stage). This indicated diffuse bilateral deep venous thrombosis of the lower extremities.

Complementary laboratory testing showed elevated D-dimers at 17,633 ng/ml. The further workup for thrombophilia, autoimmune disease, or viral disease was negative. Complementary investigations included upper digestive tract echo-endoscopy, flourine-18 fluorodeoxyglucose positron emission tomography/computed tomography, thrombophilia screening including protein S, protein C, and antithrombin deficiency, Factor V Leiden and prothrombin 20210A mutation, homocysteine plasma level, and antiphospholipid antibodies including lupus anticoagulant and anticardiolipins. Serum electrophoresis, blood flow cytometry, and autoimmune serology (Antinuclear Antibody (ANA), Anti-Neutrophil Cytoplasmic Antibodies (ANCA)) as well as viral serology for cytomegalovirus were negative.

Though structurally unconfirmed by the radiologists, several indirect clues suggested a hypoplasia of the inferior vena cava: young age, thin aspect of the suprarenal vena cava, and the presence of multiple venous collaterals. However, the thrombosis of the inferior vena cava remains idiopathic in the present case because no direct imagery abnormalities of the inferior vena cava were observed.

Final diagnosis was idiopathic inferior vena cava thrombosis extending to both legs. Treatment was initiated with therapeutic low-molecular-weight heparin (enoxaparin 6000 UI 2×/day) relayed by rivaroxaban at day 5. The patient was discharged from the hospital after a 6-day stay and recovered fully. The patient until now is following up at the outpatient clinic of internal medicine to monitor anticoagulant treatment. Therapeutic guidelines in case of unprovoked severe thrombosis in children advocate for long-term anticoagulant therapy [3]. The reason for qualifying the thrombosis as severe and unprovoked was presence of a life-threatening extensive deep venous thrombosis with all known structural and functional and inherited and acquired risk factors excluded.

## Discussion and conclusion

In literature, few similar cases are reported, with all of them having underlying causes such as external compression or pathological changes within the vein wall [4]. The particularity of the presented case is that no external cause, structural abnormality, or any systemic disease was found, predisposing the patient for a vena cava thrombosis.

Thrombosis of the inferior vena cava is a rare but life-threatening entity. It is underrecognized and associated with serious short- and long-term morbidity and mortality [5]. Increased awareness is essential because deep vein thrombosis in children seems to cause atypical symptoms, such as spinal pain or the absence of edema of the lower limbs, as in the present case.

The amount of time elapsed between the start of the symptoms and the time of diagnosis (5 weeks) is striking. Three reasons may account for this time interval: (1) the patient went to the emergency room 3 weeks after initial symptoms, (2) abdominal ultrasonography at the first Emergency Room (ER) visit was unremarkable, and (3) in Belgium, access to magnetic resonance imaging is regulated by the federal government and therefore not readily available for the emergency department [6].

To increase awareness for similar cases, the screening for red flags in acute back pain should be performed systematically and physicians should be educated on recent guidelines in the pediatric population. Specific recommendations for the diagnostic management of children with back pain are available [7–9]. These valuable guidelines summarize consensus-based expert opinions and evidence-based research, and are crucial for recognizing the broad spectrum of specific causes of back pain in children and adolescents.

Spinal pain in young patients should always trigger an elaborate clinical and diagnostic workup. It should never be considered benign. This case report emphasizes that young individuals without any previous health issues may rapidly develop a severe condition though they present initially with only back pain.

## Timeline

**8 January 2020 **Emergency room (physical examination, blood analysis, urinalysis, and abdominal echography).

**16 January 2020 **Emergency room (physical examination, referral to Dept. of Physical and Rehabilitation Medicine).

**23 January 2020 **Outpatient Clinic Physical and Rehabilitation Medicine (physical examination).

**24 January 2020 **Magnetic resonance imaging of lumbar spine. Computed tomography of abdomen. Blood analysis. Hospitalization in Department of Internal Medicine. Start of low molecular weight heparin treatment.

**29 January 2020 **Positron emission tomography with computer tomography scan (PET-CT). Endoscopic ultrasound of the upper gastrointestinal tract. Switch to rivaroxaban.

**30 January 2020 **Doppler ultrasonography of lower limbs. Discharged from hospital.

**25 February 2020 **Follow-up outpatient clinic of Internal Medicine.

**17 June 2020 **Follow-up outpatient clinic of Physical and Rehabilitation Medicine. Follow-up MRI of dorsolumbar spine.

**22 November 2021 **Magnetic resonance imaging of inferior vena cava and iliac veins.

## Data Availability

Not applicable.
